# Immune-related adverse events in immunotherapy: Challenges in diagnosis, monitoring, and management

**DOI:** 10.1016/j.toxrep.2025.102036

**Published:** 2025-04-23

**Authors:** Mohd Aftab Siddiqui, Afreen Usmani, Mohd Nazam Ansari, Rania I.M. Almoselhy

**Affiliations:** a Faculty of Pharmacy, Integral University, Lucknow, U.P., India; b MESCO Institute of Pharmacy, Amroha, U.P., India; c Department of Pharmacology and Toxicology, College of Pharmacy, Prince Sattam Bin Abdulaziz University, Alkharj 11942, Saudi Arabia; d Oils and Fats Research Department, Food Technology Research Institute, Agricultural Research Center, Giza, Egypt

**Keywords:** Immunotherapy Safety, Immune-Related Adverse Events (irAEs), Monitoring Protocols, Risk Stratification, Toxicity Management, Patient Education and Support

## Abstract

This article provides a comprehensive overview of safety and monitoring guidelines in immunotherapy, a rapidly advancing field in cancer and autoimmune treatment. Immunotherapy, which harnesses the body’s immune system to combat diseases, has shown remarkable promise but presents unique safety challenges requiring rigorous oversight. The article examines various immunotherapies, including immune checkpoint inhibitors, monoclonal antibodies, and cytokine therapies, highlighting potential adverse effects and the critical need for careful monitoring. It emphasizes pre-treatment assessments, risk stratification, and genetic and biomarker screening to identify high-risk patients. Detailed monitoring protocols, including laboratory tests, imaging, and clinical evaluations, are discussed to detect immune-related adverse events (irAEs) early. Strategies for managing both acute and chronic toxicities, such as dose adjustments and treatment interruptions, are outlined to ensure timely intervention and individualized care. The article also underscores the importance of patient education, compliance, and supportive care, emphasizing a multidisciplinary approach. Through this detailed review, healthcare professionals gain practical guidelines to optimize the safe and effective use of immunotherapies in clinical settings.

## Graphical Abstract

**Figure:**
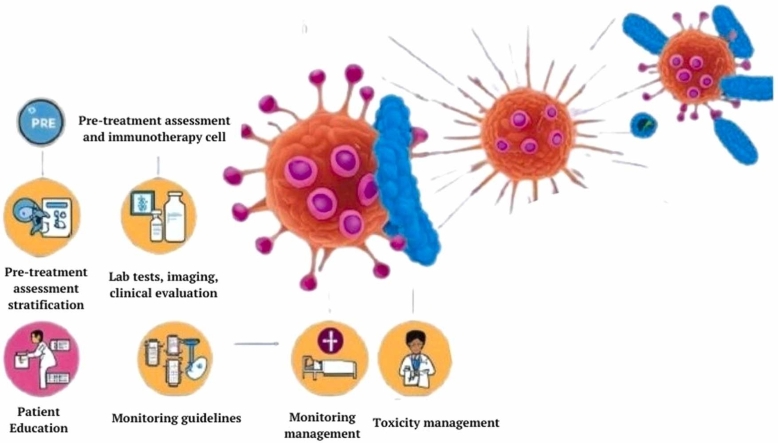

## 1 Introduction

Immunotherapy is a groundbreaking cancer treatment that uses the body's immune system to identify and attack cancer cells. It provides a more precise and often less toxic alternative to chemotherapy and radiation [1]. It involves different treatments aimed at increasing the immune response against those kinds of cells, including immune checkpoint inhibitors, monoclonal antibodies, and cancer vaccines. Targeting either a pathway or enhancing immune activity, immunotherapy provides personalized options often more effective than traditional treatments such as chemotherapy. Its indications for cancer, autoimmune, and infectious disease treatments show its great potential to reshape the future of healthcare [2], [3].

Safety and monitoring guidelines in immunotherapy are very critical because of the potential development of immune-related adverse events, which may seriously affect patient health. These adverse events, often mimicking various immune-mediated inflammatory diseases, can range from mild to severe and may require extensive treatment, including high-dose steroids to mitigate their effects [4]. Recognition and management of irAEs early are important to maintain the balance between therapeutic efficacy and patient safety. The MEDALLION study highlights that immune monitoring provides clues toward the underlying mechanisms that will be important for the prevention and management of irAE. This effort underlines the need to embed immune surveillance into the clinical routine to identify biomarkers predictive of the risk of irAE for the appropriate tailoring of interventions [4], [5]. Safety surveillance is particularly important in early-phase clinical trials, where the primary objectives are to identify safety signals and ensure participant protection. It is, therefore, important that robust pharmacovigilance frameworks be developed for the systematic identification and mitigation of potential risks [6]. ESMO has thus designed elaborate guidelines on managing iAEs, adding that it be built with the grading of these adverse events, in accordance with how treatment can best be harmonized. Guidelines by the panel assure clinicians of a more standardized approach toward diagnosing and managing treatment toxicities with follow-up for therapy toxicities, all while delivering effective care [7], [8].

The 2022 ESMO Clinical Practice Guidelines include a structured approach to diagnose, manage, and follow up on immune-related adverse events occurring with immune checkpoint inhibitors (ICIs). Recommendations regarding toxicity grades of intensity are as follows: mild (Grade 1)-symptom monitoring may be done; treatment may continue. Moderate to Severe (Grades 2–4): ICI interruption, corticosteroid therapy, and in refractory cases, advanced immunosuppressive treatments (e.g., infliximab) [7]. Real-world studies demonstrated shortcomings in toxicities management. For example, guideline-concordant management for 47 % of irAEs shows there is a possibility for improvement regarding documentation, education, and implementing best practices into routine care [9]. More modern ways, like nurse-led follow-up and active monitoring programs, tend to have great potential and be increasingly effective in developing a more patient-centered culture of care. These tools enhance the chances of early intervention while offering a better quality of life by preventing or moderating therapy-related symptoms [10]. This needs to be applied at a routine level with full access to multidisciplinary supportive care.

## 2 Immunotherapy in cancer and autoimmune diseases

**Fig. 1:**
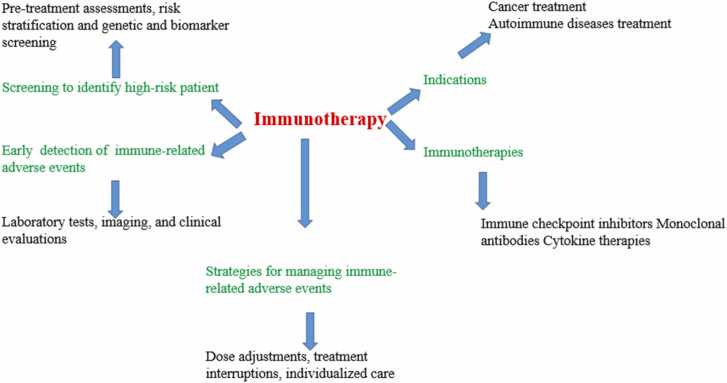
Key aspects of immunotherapy: screening, treatment, and management.

Immunotherapy in terms of cancer and autoimmune treatment involves a few strategies that have so far been laid down to take advantage of the immune system in fighting malignancies while causing minimal side effects, steps are shown in Fig. 1. Among them, ICIs targeting CTLA-4, PD-1, and programmed death-ligand 1 (PD-L1) have revolutionized the treatment of cancer by abrogating inhibitory pathways that prevent T cells from attacking tumors. While these therapies have demonstrated significant efficacy in the treatment of advanced malignancies, there are challenges like resistance to treatment and immune-related adverse events [11], [12]. Then came chimeric antigen receptor T-cell therapy, especially in hematologic malignancies, offering durable responses in otherwise refractory cases. Similarly, whole-tissue autologous therapeutic vaccines represent a novel approach whereby the use of a patient's own tumor tissue is utilized to stimulate an immune response without inducing autoimmunity or cancer seeding. Systematic reviews have shown minimal risks associated with WATVs, underlining their safety profile [13]. It ensures the efficacy of these therapies through personalized treatment plans for each individual according to genetic and molecular profiles. A personalized approach reduces adverse events, thus making precision medicine all the more critical in immunotherapy [14].

Emerging areas of research include the coupling of immunotherapy with nanoparticles to enhance drug delivery and efficacy, as well as the adaptation of other immune cells, such as natural killer (NK) cells and macrophages, to broaden therapeutic applications. These innovative strategies address the limitations of existing therapies and explore mechanisms to overcome resistance [15]. Further, the identification of newer immune checkpoint targets, such as TIGIT and TIM-3, expands further opportunities for combination therapies, which can lead to improved responses and reduced resistance in patients' outcomes [16].

## 3 Immune-related adverse events and safety considerations

IrAEs have a great impact on the outcome of treatment, especially in patients with a predisposition to autoimmune diseases; therefore, careful selection and follow-up of patients should be performed. The acquired knowledge of irAEs is also translated into the strategy of their management in order to make sure that safety is adequately counterbalanced with therapeutic efficacy. The role of monoclonal antibodies (mAbs) extends beyond checkpoint inhibition to include therapies targeting specific tumor antigens or modulating immune responses. These versatile agents have demonstrated efficacy in both cancer and autoimmune diseases, solidifying their role as a cornerstone of immunotherapy [17]. Indeed, newer modalities include bispecific antibodies directed against two different antigens and cytokine therapies consisting of interleukins and interferons that extend the armamentarium continually [16], [18]. Additionally, ICIs have been particularly impactful in the treatment of advanced non-small cell lung cancer (NSCLC), where therapies targeting CTLA-4, PD-1, and PD-L1 have shown promise. Recent advancements in monoclonal antibody development are further enhancing therapeutic options for this challenging context [19].

## 4 Safety challenges in immunotherapy

Immunotherapy has emerged as a game-changing strategy in cancer and autoimmune disease treatment, leveraging the immune system's capabilities to target malignant cells and control aberrant immune responses. Modalities include monoclonal antibodies, immune checkpoint inhibitors, and CAR-T cell therapies; each has proven to be highly effective in various malignancies by increasing immune recognition and tumor destruction [17], [18], [19]. Although clinical trials show promising results, immunotherapy's effectiveness is often limited by factors such as the tumor microenvironment, cancer cells' immune evasion tactics, and tumor antigen variability. Overcoming these challenges requires new strategies [20].

### 4.1 Immune-related adverse events (irAEs)

irAEs are one of the leading safety concerns in immunotherapy and result from the increased activity of the immune system against healthy tissues. These events can range from mild to very serious and manifest differently, including dermatitis and colitis, which usually respond well to supportive care and corticosteroids. The most severe cases may present life-threatening conditions, including myocarditis, neurological complications, or cytokine release syndrome hyperinflammatory state triggered by excessive cytokine production [21], [22], [23]. The underlying mechanisms for irAEs are multifaceted and complex; however, they are believed to arise from the breach of immune tolerance. This is particularly seen with ICIs that involve anti-CTLA-4 and anti-PD-1/PD-L1 agents because they remove the inhibitory checkpoints on immune activity [24].

### 4.2 Common adverse effects and risk factors

The risk factors will vary depending on the subject characteristics, treatment combinations, or specific agents used. Such is the case when combining ICIs, such as anti-CTLA-4 and anti-PD-1 therapies: the incidence of severe irAEs is higher than observed with monotherapy. Or, for example, where certain agents are associated with select toxicities, including the neurological complications seen with PD-1 inhibitors [22].

### 4.3 Determinants of safety outcomes

Management of irAEs is usually multidisciplinary, often involving the oncologist, immunologist, and specialist for specific organs.

## 5 Various aspects in the management of irAEs

a. **Diagnosis and Treatment:** Early recognition of the disease is essential since delays in diagnosis are associated with greater severity. Treatment generally consists of corticosteroids for immune suppression, although in refractory cases, other immunosuppressive agents such as infliximab or mycophenolate mofetil are added [24], [25].
b. **Lack of FDA-Approved Treatments:** No specific therapies for irAEs have received FDA approval to date, complicating standardized care. This further underlines the need for new treatment options and clinical guidelines with a sense of urgency [21], [22].
c. **Predictive Biomarkers:** Biomarker development to predict the risk for irAEs is an active area of research. Biomarkers may enable pre-emptive strategies that reduce the incidence and severity of these events [21].

## 6 Pre-treatment assessment and risk stratification

Pre-treatment assessment and risk stratification are the first moves to guarantee that the level of immunotherapy will be safe and effective. Pre-treatment assessment allows for screening of the best patients most likely to benefit from treatment with the potential patients at an increased risk for adverse side effects. Key components entail patient assessment, baseline workup, high-risk identification, and genetic/biomarker testing.

### 6.1 Patient evaluation and baseline testing

Patient assessment and baseline testing are also important in the preparation prior to immunotherapy [26]. The detailed physical and medical history evaluates her actual and past conditions; conditions such as autoimmune disorders and infections, including dysfunctions of organs are discussed with her. Reference blood examinations include complete blood counts; functions of the liver, the kidneys, and all markers of inflammation like the one known as CRP levels. Imaging techniques comprise scanning by CT, MRI, or PET in calculating disease burden and assessing an optimal response to therapy in later stages. For patients with a history of cardiovascular issues, baseline evaluations such as electrocardiograms (ECG) or echocardiography may be necessary to assess cardiac function [27].

### 6.2 Identification of high-risk patients

Identifying high-risk patients is critical to prevent adverse outcomes during immunotherapy [28]. Factors such as advanced age, multiple comorbidities, and pre-existing organ dysfunction increase the likelihood of complications. Patients with autoimmune diseases require special consideration, as immunotherapy may exacerbate these conditions. A systematic functional capacity test is done via appropriate performance status tools such as the Eastern Cooperative Oncology Group or Karnofsky scale, and all current medications for potential drug- immunosuppressives and/or steroids are listed; the presence of psychosocial risks is assessed because this risk factor impacts their ability to adhere to, manage the treatment and has implications at the psychosocial, social, financial, occupational, and interventional realms [29].

### 6.3 Genetic and biomarker screening

Genetic and biomarker screening plays a pivotal role in predicting treatment response and potential toxicity risks in immunotherapy [30]. Biomarkers such as PD-L1 expression and tumor mutational burden (TMB) provide critical insights into a patient's likelihood of responding to immune checkpoint inhibitors. Human leukocyte antigen (HLA) typing can help predict susceptibility to severe irAEs, while germline mutation analysis (e.g., BRCA, mismatch repair (MMR) genes) is particularly useful for identifying hereditary cancer risks [30]. Baseline cytokine profiling is emerging as a tool for assessing the risk of immune hyperactivation, including complications like cytokine release syndrome (CRS). Pharmacogenomic testing is also warranted to identify genetic variations with a significant impact on drug metabolism and/or immune response pathways [31].

## 7 Monitoring protocols for irAEs

Recent studies, including the MEDALLION trial, highlight the importance of immune monitoring in predicting and preventing irAEs. The study demonstrated that real-time biomarker tracking improved early intervention rates, reducing severe toxicity incidence by 30 % [4], [5]. Similarly, ESMO guidelines now recommend integrating standardized monitoring protocols based on real-world patient outcomes, which have shown improved adherence to toxicity management guidelines [6], [7]. Immune-related adverse events monitoring protocols are significant in patients treated with ICI therapy. A multifaceted approach that incorporates laboratory testing, imaging techniques, and clinical assessment for the early detection of most irAEs is of paramount importance since their nature is unpredictable and often severe. An integrated approach helps to maximize the benefit-to-risk profile in patients [31].

### 7.1 Laboratory tests

Laboratory tests are essential in the diagnosis of the onset and severity of irAEs. These usually include comprehensive tests of the components of the immune system and biomarkers, which can give an early indication of immune dysregulation. Immune cell phenotyping enables the monitoring of changes in immune cell populations that can signal the activation of autoimmunity. Serum cytokine profiling can highlight the change in immune response, and some cytokines may be raised in the presence of irAEs, including IFNγ. Moreover, the monitoring of the neutrophil-to-lymphocyte ratio has been inversely related to the grade of immune-related adverse events [32].

These biomarkers provide tools for early intervention and give the clinician the opportunity to modify the treatment approach before the appearance of life-threatening irAEs.

### 7.2 Imaging techniques

While laboratory assessment is important, the utilization of advanced imaging is what provides critical information in assessing tumor dynamics and treatment responses in real-time. Various imaging techniques, including PET and MRI, are commonly used for observing the changes of the tumor and other systemic immune-mediated effects on nontarget organs. Liquid biopsies offer a novel approach by enabling the detection of circulating tumor cells and other biomarkers in the bloodstream, providing continuous monitoring of the tumor’s molecular characteristics and any shifts in immune function [24]. These non-invasive methods provide a dynamic picture of treatment efficacy and immune system activity.

### 7.3 Clinical evaluations

These assessments, including physical examination and symptom monitoring, are equally crucial in the diagnosis of irAEs, which can be quite nonspecific in many organ systems. Routine symptom monitoring, such as symptoms affecting the skin, gastrointestinal tract, and respiratory system, allows for early detection of subtle signs of side effects of treatment early in the treatment cycle. Physical examinations and a full review of patient-reported outcomes provide much information on the clinical status of the patient that may not always be picked up through investigations of laboratory or imaging tests alone [22], [33].

### 7.4 Integration of monitoring strategies

The integration of laboratory tests, imaging techniques, and clinical evaluations into a coordinated monitoring strategy allows the optimum management of all the complexities associated with irAEs for the attainment of best patient outcomes. Early identification and timely management prevent the progression to severe or irreversible damage. Additionally, a personalized approach that tailors the intensity and frequency of monitoring to the patient’s specific risk factors, treatment regimen, and organ systems involved is key to improving the safety and efficacy of immunotherapy [33].

## 8 Managing adverse events in immunotherapy

Immunotherapy should be managed through the lens of both acute and chronic toxicities. Recognizing that irAEs may become severe, they need to be promptly noticed and addressed to ensure both the safety and maximum benefit of the patient.

### 8.1 Acute toxicities

The acutely toxic periods of immunotherapy are commonly of more immediate concern due to the fact that they may rapidly escalate and need intervention in an extremely short time. The most serious toxicities are immune-mediated pneumonitis and myocarditis, which must be identified promptly. Treatment of grade II or higher toxicities usually consists of corticosteroids, which dampen the immune response and prevent further tissue injury. In grade I toxicities, for example, mild pneumonitis, treatment interruption is not necessary, but then again, symptoms must not be allowed to deteriorate [34], [35]. The challenge in the management of acute toxicities is the balance between early use of immunosuppressive treatments and maintaining the efficacy of immunotherapy. Early recognition and timely interventions reduce the risk of serious complications significantly.

### 8.2 Chronic toxicities

Chronic toxicities, although a bit less immediate, can set up effects on the health and quality of life of the patient. One of the most alarming long-term toxicities has to do with nephrotoxicity: the immune-mediated renal adverse events requiring long-term management and monitoring. These include immune-mediated glomerulonephritis or acute kidney injury and other renal manifestations. The importance of long-term follow-up studies play an important role in the early detection of changes in renal function-the early action that can avoid irreversible changes. Kidney biopsies can be indicated in cases requiring confirmation of the etiology and determining the approach of care. Additionally, the determination of the risk-benefit ratio for continuing with immunotherapy after the resolution of irAEs involving the kidney must be done carefully. Moreover, clinicians must deliberate whether re-challenge following the recovery of kidney-related irAEs is feasible or beneficial [17].

### 8.3 Personalized treatment plans

Development of personalized treatment plans in the management of iRAEs is underlined, since each patient can develop a different spectrum of toxicities with a questioned risk-benefit ratio of the continuation of immunotherapy. The continuation or adjustment of treatments needs to be considered based on patient-specific factors such as age, comorbidities, and history of adverse reactions. Secondly, patients' preference for continuation of therapy along with the possibility of long-term effects needs to be included into decision-making also [34]. This ensures personalized treatment according to the values of the patient and simultaneously manages the adverse effects.

### 8.4 Multidisciplinary approach

The management of irAEs requires a multidisciplinary approach to involve oncologists, immunologists, rheumatologists, and nephrologists in view of the different organ systems that may be involved. This is a multidisciplinary effort in which management of adverse events would be comprehensive, with various specialists' contributions regarding the most appropriate measure for each individual patient [17]. As certain toxicities are chronic, communication and coordinated care are part of the holistic treatment to avert complications. Overall, AEs with immunotherapy need timely responses to acute toxicities, long-term follow-up in cases of chronic toxicities, and tailored treatments based on both clinical and patient-centered factors.

## 9 Patient education and compliance in immunotherapy

A systematic review of immunotherapy safety trials revealed that while checkpoint inhibitors offer substantial survival benefits, approximately 47 % of patients do not receive guideline-concordant toxicity management, highlighting the need for improved compliance strategies [9]. Real-world studies have demonstrated that nurse-led follow-up programs can reduce the incidence of high-grade irAEs by 25 %, underscoring the role of structured patient support in improving treatment adherence [10]. Patient education and compliance are significant to successful immunotherapy management and have a direct influence on both treatment outcomes and quality of life. Several studies indicate that a large number of patients abandon their immunotherapy for various barriers such as complicated treatment regimens, high treatment costs, and adverse reactions. These challenges underscore the need for effective patient education strategies to support adherence and enhance therapeutic efficacy [36], [37].

### 9.1 Enhancing patient understanding of immunotherapy

Educational tools that provide patients with a thorough understanding of potential irAEs are essential in empowering them to recognize early symptoms and take proactive measures. Education can play a very important role in the prevention of treatment interruptions and enhancement of adherence by informing patients about the risks and signs of irAEs. It has been established that patients educated on potential side effects from immunotherapies report symptoms earlier in the process and adhere more strongly to treatment adjustments, hence minimizing the frequency and severity of adverse events [38]. Furthermore, individualized education on specific individual patient needs and awareness is paramount. Particular topics that concern them, such as the management of symptoms related to therapy and possible long-term effects, make them feel more in control of their treatment process. This may lead to an enhanced capability in making informed decisions and increased compliance with the prescribed regimen.

### 9.2 Supportive care and empathetic communication

Besides education on treatment and possible adverse events, personalized supportive care is important for improving patient adherence and outcomes. Models like the Subjective Health Experience Model underline the importance of empathetic communication and the need to tailor interventions to meet the individual needs of each patient. By understanding and addressing the unique challenges patients face—whether physical, emotional, or psychological—clinicians can provide more effective support, which in turn fosters better patient engagement and long-term adherence to immunotherapy [39]. Effective patient support programs should combine educational, motivational, and communication strategies to actively engage patients. These programs can help patients feel supported throughout the treatment process and better equipped to manage the complexities of immunotherapy, thereby improving treatment adherence and long-term outcomes.

### 9.3 Strategies to improve compliance

The nature of patient compliance requires an all-encompassing approach to patient education beyond one-time sessions. Individualized education sessions, written materials, and continued support networks are some of the methods through which information can be reinforced to prepare patients for the challenges in treatment. Access to resources for the patients, such as support groups, mobile applications, or 24/7 consultation services, can easily resolve issues as they arise, leading to better compliance and fewer discontinuations in therapy.

Moreover, studies have evidenced that the use of standardized teaching tools greatly enhances patients' understanding both of treatment approaches and potential side effects during immunotherapy. This is especially poignant in cases such as that of triple-negative breast cancer, where these educational interventions promoted more patient involvement and properly informed consent, thus fostering compliance with treatments and symptom improvement [37].

In the context of sublingual allergen-specific immunotherapy, this very principle has been applied in strategies known to enhance compliance and minimize dropouts. Several investigations reported that a visit-to-visit management able to update education can facilitate patient knowledge of salient treatment facts. Addressing perceptions of improved illness, reduced symptomatic medication enhanced adherence to SLIT among children [38].

### 9.4 Overcoming barriers and enhancing health literacy

Effective patient education in immunotherapy takes into consideration health literacy, addresses misinformation, and facilitates treatment adherence. Healthcare providers should measure patients' knowledge about treatment principles and risks and the rationale for treatment adherence. The involvement of the patient in decision-making and assurance that treatment options have been understood and an opportunity to ask questions will enhance the chances of compliance with treatment. Furthermore, debunking misinformation reduces anxiety about the treatments and prevents decisions based on misconceived notions. These implemented strategies will greatly enhance patient engagement, with consequent improved outcomes and compliance. Patients informed about their immunotherapy, its possible side effects, and the need for consistent treatment are better positioned to navigate their therapy successfully and remain compliant in the long term [38].

### 9.5 Hospital-based patient education programs

Several cancer centers and hospitals worldwide have developed comprehensive patient education programs to support individuals undergoing immunotherapy. These initiatives have demonstrated significant success in improving treatment adherence, patient engagement, and irAE management. One such example is the Memorial Sloan Kettering Cancer Center’s Immunotherapy Education Program, which provides personalized education sessions for patients before and during treatment. These sessions focus on understanding immunotherapy mechanisms, recognizing early signs of irAEs, and managing side effects proactively. Patients who participated in this program were 30 % less likely to discontinue treatment prematurely due to manageable toxicities [38].

Similarly, The Mayo Clinic's Multidisciplinary Immunotherapy Education Initiative combines oncologist-led counseling, nurse navigator guidance, and digital education platforms to provide ongoing patient support. Their study found that patients who received structured education had a 25 % reduction in emergency department visits for irAEs and demonstrated improved self-reporting of early symptoms, leading to timelier interventions [38]. A recent analysis of the MD Anderson Cancer Center’s Patient Engagement Model revealed that educating patients about immune-related toxicities increased compliance rates by 40 %, especially among patients undergoing immune checkpoint inhibitor therapy. The initiative provided individualized risk assessment sessions, digital monitoring tools, and a 24/7 helpline for symptom management, significantly reducing hospital admissions related to treatment complications [38], [39].

## 10 Multidisciplinary approach to immunotherapy administration and management

The multidisciplinary approach remains indispensable for safe administration and management, especially with the complex challenge that immune-related adverse events present. While immunotherapies, especially via ICI, have changed the face of cancer treatment, they also present new toxicities, requiring a broad skill set in the management of health professionals. Such teamwork between oncologists, pharmacists, nurses, and other professionals ensures timely detection and effective treatment of the onset, progress, and resolution of irAEs [40], [41].

### 10.1 Rationale for interprofessional collaboration

The nature of managing irAE requires expertise and views from a multidisciplinary team approach. Most treatment decisions are led by oncologists, but they need the pharmacists' consultation regarding certain drug interactions, possible toxicities, and symptomatic medications. The nurses will have to monitor the patients very closely, report the first signs of toxicity, and make sure that the patients follow the treatment regimens. With better collaboration among professionals, potential adverse reactions would be timely identified and reduced, allowing early intervention in the prevention of greater risks for the patient. Moreover, collaboration allows healthcare providers to share insights and optimize therapeutic strategies, improving both patient safety and outcomes [42].

### 10.2 Education and communication

The mainstay of irAE management involves good communication and coordination between the healthcare providers. Ongoing training through structured educational programs regarding the latest protocols on immunotherapy and toxicities is important to keep all team members informed. These programs are critical to equipping providers with the capability to identify irAEs early and to respond appropriately. Furthermore, the use of telemedicine platforms facilitates real-time communication and consultation, allowing healthcare teams to collaborate efficiently, even when they are not physically in the same location [43].

### 10.3 The role of nursing teams

Nurses play an important role in monitoring and managing irAEs. They have to execute regular evaluations, early warning toxicity signs, and follow the care protocol. These often consist of administering medications that deal with side effects and the alleviation of symptoms and instructing patients on the management of adverse effects. Other responsibilities of a nurse are educating the patients on possible side effects brought about by immunotherapy and the need for timely reporting of the symptoms [42].

For instance, Galioto et al. [42] established how, through nurse-led monitoring and follow-up, patient outcomes are significantly enhanced to ensure that symptoms are identified much earlier to be addressed as soon as possible and, therefore, reducing the duration and intensity of iAEs. This supports how nurses are significant in the everyday management of patients undergoing immunotherapies as a first line of defense in symptom finding and intervention.

### 10.4 Creating a cohesive supportive framework

Overall success for immunotherapy depends on such a cohesive, multidisciplinary framework of clinical acumen, communication, and focus on the patient. By collaborating across disciplines, healthcare professionals develop an enabling environment that enables not only better management of irAEs but also enhancement of patient care. By doing so, this very collaborative framework will go a long way in reducing overall toxicities, preventing certain complications, and thereby ensuring adherence and quality of life among patients undergoing immunotherapy [41].

## 11 Integration of digital health technologies in immunotherapy

Digital health technologies are rapidly transforming the safety and monitoring of immunotherapy, offering promising solutions for the early detection and management of immune-related adverse events (irAEs). Wearable devices, AI-powered tools, and remote monitoring systems are now increasingly integrated into clinical practice, improving real-time patient monitoring and early intervention. These advancements are particularly valuable for detecting cardiotoxicity, pneumonitis, cytokine release syndrome (CRS), and other immune-related complications, allowing for timely therapeutic adjustments and reduced hospitalizations [44].

### 11.1 Wearable devices and remote monitoring

Wearable technologies, including smartwatches, biosensors, and wireless patches, have become valuable tools for the continuous monitoring of patients undergoing immunotherapy. These devices track vital signs such as heart rate, blood pressure, oxygen saturation, and body temperature, providing early warning signs for immune-mediated complications. In a real-world study, continuous ECG monitoring in patients receiving ICIs successfully identified early arrhythmias and myocarditis, reducing severe cardiac events by 40 % [45]. Similarly, wearable biosensors tracking inflammatory markers were able to predict immune-mediated colitis and hepatitis days before symptom onset, allowing early corticosteroid intervention and improving patient outcomes [45]. Advanced wearable sensors now incorporate skin temperature and hydration monitoring, which helps detect early signs of immune-mediated dermatitis or fever, both of which are common early indicators of irAEs. These real-time physiological data are transmitted to healthcare providers, enabling proactive risk assessment and timely intervention before symptoms worsen. Additionally, wearables track patient activity levels, providing insight into fatigue and systemic inflammatory responses—common indicators of subclinical immune toxicity in immunotherapy [45].

Wearable devices and remote monitoring face challenges despite their potential benefits. Accuracy issues arise due to sensor sensitivity, improper placement, and external factors affecting data reliability. Patient compliance remains a concern, as device maintenance, discomfort, and battery life constraints can hinder long-term usage. Additionally, integrating wearable data into clinical workflows poses technical and logistical difficulties, requiring seamless interoperability with existing healthcare systems [46].

### 11.2 Artificial intelligence and machine learning in immunotherapy monitoring

Artificial intelligence (AI) and machine learning (ML) have demonstrated significant potential in identifying early toxicity markers and predicting irAE severity based on clinical data patterns. AI models analyze immune cell counts, cytokine fluctuations, and biomarker trends to forecast high-risk patients before symptoms appear. A 2024 clinical study found that AI-driven machine-learning models analyzing immune cell counts and cytokine profiles could predict severe irAEs with 85 % accuracy, allowing for early therapeutic intervention [47]. Another study demonstrated that AI-assisted symptom monitoring reduced high-grade toxicity events by 30 %, showcasing the power of predictive analytics in immunotherapy safety. Moreover, AI is improving radionics-based analysis, where machine-learning algorithms extract imaging biomarkers from CT and MRI scans to detect early signs of organ inflammation due to irAEs. In one study, AI-enhanced imaging detected subtle signs of immune-related pneumonitis weeks before clinical symptoms appeared, leading to earlier corticosteroid initiation and improved patient survival rates [47]. AI-powered genomic risk stratification is also being developed to assess individual patient susceptibility to irAEs. By integrating genomic data with clinical records, AI algorithms can personalize treatment regimens, reducing toxicity risks while maintaining therapeutic efficacy. These precision medicine approaches are revolutionizing immunotherapy patient management [47].

AI and machine learning in immunotherapy monitoring also have limitations. Data bias is a significant challenge, as AI models rely on diverse datasets, and disparities in data collection can impact generalizability. Transparency issues arise because many AI models function as "black boxes," making clinical validation and trust in decision-making difficult. Furthermore, regulatory approval remains a hurdle, requiring extensive validation and oversight to ensure the reliability and safety of AI-driven diagnostics [48].

### 11.3 Digital health integration in clinical settings

Emerging research suggests that AI-powered biomarker screening and digital health integration could revolutionize irAE management by detecting predisposing genetic signatures and enabling real-time monitoring. A recent clinical trial demonstrated that integrating AI-driven predictive models into immunotherapy safety protocols reduced treatment-related mortality by 18 %, underscoring the role of precision medicine in toxicity prevention [49], [50]. Additionally, telemedicine platforms are improving remote patient care, allowing for virtual consultations and real-time symptom monitoring. Patients using telemedicine-assisted symptom tracking experienced a 35 % reduction in emergency hospitalizations due to immunotherapy-related toxicities, demonstrating the effectiveness of remote intervention strategies [49]. The integration of cloud-based digital health platforms allows patients to upload wearable device data for review by healthcare providers, enabling ongoing monitoring and real-time intervention when necessary. AI-driven prediction models further enhance risk assessment, helping clinicians optimize follow-up schedules and adjust therapy based on patient-specific risks [49], [50].

Digital health integration in clinical settings faces obstacles related to data security, interoperability, and accessibility. Cloud-based platforms raise concerns about patient privacy, with risks of unauthorized access and cyber threats. Ensuring seamless integration with existing healthcare infrastructure remains a challenge, particularly for institutions using outdated electronic health records. Moreover, accessibility to AI-driven telemedicine services is limited in rural and underserved areas due to internet connectivity issues and varying levels of technological literacy, potentially widening healthcare disparities [51].

## 12 Safety of immunotherapy: a global perspective

Immunotherapy has significantly transformed cancer treatment; however, its complexity necessitates robust safety monitoring to manage irAEs. The regulatory frameworks, healthcare infrastructure, and cultural attitudes towards immunotherapy vary globally, complicating uniform safety standards.

### 12.1 Regulatory frameworks

Regulatory bodies like the US FDA and European Medicines Agency enforce stringent safety measures for immunotherapy drugs, focusing on pre- and post-approval monitoring. In India, the Central Drugs Standard Control Organization (CDSCO) oversees drug approvals, but challenges persist in standardizing safety protocols, particularly in rural areas where resources are limited [52].

### 12.2 Healthcare infrastructure

Advanced oncology centers benefit from comprehensive resources, including biomarker testing and trained specialists, facilitating effective irAE management. While urban centers are working on adopting advanced technologies, the rural healthcare system fails to diagnose or provide treatment facilities that could address the growing burden of malignancies [53].

### 12.3 Cultural and social influence

Low health literacy observed among the population in India often leads to late reporting and interference in taking timely action. Traditional practices might be prevailing and influencing patients' acceptance of immunotherapy [53].

## 13 Future approaches in immunotherapy

Researchers are working to identify new biomarkers, refine patient selection, and develop combination treatments to boost immunotherapy effectiveness while reducing side effects.

### 13.1 Personalized and precision immunotherapy

Utilizing artificial intelligence and machine learning to dissect individual patient-specific data, as well as predict potential response, could lead to precisely designed regimens maximizing their efficacy and minimizing their toxicities. Immune checkpoint inhibitors show remarkable results in some patients, but response rates vary, and many do not benefit. This underscores the need for predictive biomarkers and personalized treatments [54].

### 13.2 Next-generation therapies

CAR-T therapy for cancers beyond hematological malignancies, the development of oncolytic viruses, and the tailoring of neoantigen vaccines present promising next-generation immunotherapy approaches. Combining immunotherapy with chemotherapy, targeted therapy, or radiation is being explored to improve effectiveness and overcome treatment resistance. Advances in immunotherapy, especially immune checkpoint inhibitors and adoptive cell transfer, have revolutionized cancer treatment, giving hope to patients with advanced or resistant cancers [55].

### 13.3 Advancements in biomarker development

Dynamic biomarkers reflecting real-time changes in the immune response can be better detected with advanced techniques like liquid biopsies and imaging [56].

### 13.4 Overcoming healthcare disparities

Expanding telemedicine integration and promoting global collaboration can increase access to immunotherapy in low-resource settings. It is important that data sharing and best practice exchange are done across borders for equitable access to treatment [56].

## 14 Conclusion

Immunotherapy is a continuously evolving field, and because of that, it has totally revolutionized cancer and autoimmune disease management by providing better results for patients. On the other hand, this new development opens many challenges in different aspects mainly regarding safety, monitoring, and managing patients. Because of the broad varieties of immunotherapies such as immune checkpoint inhibitors, CAR-T cell therapy, and autologous therapeutic vaccines, the concept of personalized medicine gained even greater momentum. These are indeed transformative therapies but equally accompanied by immune-related adverse events that demand keen vigilance for their early detection and timely intervention. It enables a multidisciplinary approach whereby oncologists, nurses, pharmacists, and other healthcare professionals have their place in managing adverse events, further enhancing the effectiveness of immunotherapy. With improving digital health technologies, wearables, and AI-powered tools, exciting opportunities lie ahead to advance early detection and management of irAEs, placing technology even more at the forefront of healthcare. However, global variability in safety monitoring and guidelines reflects regional disparities in health care and calls for unified protocols that can provide a certain standard and equitable quality of care. Pretreatment assessments, genetic screening, and strategies regarding patient education are highly relevant and important in optimizing outcomes, improving adherence among patients, and minimizing risks. To sum up, it will take more research, innovation, and collaboration to fine-tune the safety profile of immunotherapy, surmount the existing limitations, and make the benefits of these therapies equally accessible to all patients, regardless of geographical or healthcare system considerations.

## Author statement

We, the undersigned authors, confirm the following:

1. All listed authors have made substantial contributions to the conception, design, writing, and critical revision of the manuscript. All authors have read and approved the final version of the manuscript and agree to its submission.
2. This manuscript is original and has not been published previously, nor is it under consideration for publication elsewhere. It does not infringe upon any copyright or intellectual property rights.
3. The authors declare that there are no conflicts of interest regarding the publication of this manuscript.
4. No specific funding was received for this work.
5. This manuscript does not involve any studies with human participants or animals performed by any of the authors.
6. All authors agree to be accountable for all aspects of the work, ensuring that questions related to the accuracy or integrity of any part of the work are appropriately investigated and resolved.
7. Dr. Mohd Aftab Siddiqui has been designated as the corresponding author and is responsible for all communication with the journal during the manuscript submission, peer review, and publication process.

## CRediT authorship contribution statement

**Almoselhy Rania I.M.:** Writing – review & editing, Formal analysis. **Ansari Mohd Nazam:** Writing – review & editing, Formal analysis. **Usmani Afreen:** Writing – review & editing, Writing – original draft. **Siddiqui Mohd Aftab:** Writing – review & editing, Writing – original draft, Conceptualization.

## Declaration of Competing Interest

The authors declare that they have no known competing financial interests or personal relationships that could have appeared to influence the work reported in this paper.

## Data Availability

Data will be made available on request.
